# Von Willebrand Factor Multimers and the Relaxation Response: A One-Year Study

**DOI:** 10.3390/e23040447

**Published:** 2021-04-10

**Authors:** Carlo Dal Lin, Laura Acquasaliente, Sabino Iliceto, Vincenzo De Filippis, Giuseppe Vitiello, Francesco Tona

**Affiliations:** 1 Department of Cardiac, Thoracic and Vascular Sciences, Padua University School of Medicine, Via Giustiniani 5, 35131 Padua, Italy; sabino.iliceto@unipd.it (S.I.); francesco.tona@unipd.it (F.T.); 2 Department of Pharmaceutical and Pharmacological Sciences, Padua University School of Medicine, Via Marzolo 2, 35100 Padua, Italy; laura.acquasaliente@unipd.it (L.A.); vincenzo.defilippis@unipd.it (V.D.F.); 3 Department of Physics “E.R. Caianiello”, Salerno University, Via Giovanni Paolo II, 132, 84084 Fisciano, Italy; vitiello@sa.infn.it

**Keywords:** cardiovascular disease, inflammation, von Willebrand factor, carbonyl content, endothelial function, relaxation response

## Abstract

Background and aim: Mental stress represents a pivotal factor in cardiovascular diseases. The mechanism by which stress produces its deleterious ischemic effects is still under study but some of the most explored pathways are inflammation, endothelial function and balancing of the thrombotic state. In this scenario, von Willebrand factor (vWF) is a plasma glycoprotein best known for its crucial hemostatic role, also acting as key regulatory element of inflammation, being released by the activated vascular endothelium. Antistress techniques seem to be able to slow down inflammation. As we have recently verified how the practice of the Relaxation Response (RR), which counteracts psychological stress, causes favorable changes in some inflammatory genes’ expressions, neurotransmitters, hormones, cytokines and inflammatory circulating microRNAs with coronary endothelial function improvement, we aimed to verify a possible change even in serum levels of vWF. Experimental procedure: We measured vWF multimers and the total protein carbonyl contents in the sera of 90 patients with ischemic heart disease (and 30 healthy controls) immediately before and after an RR session, three times (baseline, 6 months, 12 months), during a one-year follow-up study. Results: According to our data, large vWF multimers decrease during the RR, as does the plasma total carbonyl content. Conclusion: vWF levels seem to vary rapidly between anti-inflammatory and antithrombotic behaviors dependent on psychological activity, leading to relaxation and also possibly changes in its quaternary structure.

## 1. Introduction

Stress appears to be the basis of many diseases [1], especially cardiovascular ones. One of the most studied mechanisms at the basis of these findings is vascular psychologic (“sterile”) driven inflammation. Mental stress is accompanied by endothelial disfunction and by a prothrombotic state [2].

Von Willebrand factor (vWF) is a large plasma glycoprotein released as ultra-large-vWF (ULvWF) polymers from vascular endothelial cells and megakaryocytes and is responsible for the initiation of primary hemostasis, inflammation, and apoptosis [3,4]. vWF mediates the adhesion of platelets to sites of vascular damage by binding specific platelet membrane glycoprotein (GpIbα) and constituents of the exposed connective tissue. Hemostasis depends on the balanced participation of vWF, which reflects competition between the biosynthesis of ULvWF and their degradation by the ADAMTS-13, a metalloproteinase with thrombospondin motifs. The prothrombotic potential of vWF is the result of a dynamic equilibrium between the concentration of ULvWF and the proteolytic activity of ADAMTS-13. Once in the bloodstream, ULvWFs with a half-life of 12–40 h [5,6] are cleared by a mechanism that may not depend strongly on multimer size [7]. At the same time, ULvWFs are converted into smaller species by ADAMTS-13, which specifically cleaves the Tyr1605-Met1606 bond in domain A2 when the vWF multimer is subjected to sufficient fluid shear stress [8,9,10]. In the case of inflammation or stress, a biochemical defence mechanism releases reactive oxygen species (ROS) that can selectively oxidize amino acid residues. The oxidation of Met1606 hinders vWF cleavage by ADAMTS-13, increasing the accumulation of UL-vWF, which is more prothrombotic than short vWF oligomers. The resulting accumulation of UL-vWF polymers allows the recruitment and activation of platelets, thus contributing to the development of thrombotic complications.

Recently, we followed patients with ischemic heart disease and healthy volunteers for one year after having taught them the Relaxation Response (RR) as part of a 4-day rational-emotional-education intervention, and we documented a significant decrease in the perceived degree of stress combined with a clinically favorable variation of different neuroendocrine-immune markers of inflammation, oxidative stress [11], circulating microRNAs [12] along with coronary endothelial function improvement.

Given the introduced premises, we asked ourselves if the RR practice could be linked to possible changes in serum levels of vWF multimers and proteins’ carbonyl contents.

## 2. Materials and Methods

### 2.1. Biological Samples

We collected serum samples of 120 subjects following an approved protocol (Comitato Etico per la Sperimentazione Clinica-Azienda Sanitaria di Padova; protocol number 3487/AO/15—13/7/2015 updated number 4895/AT/20—23/7/2020) [11]. Briefly, we enrolled 90 consecutive patients after myocardial infarction and 30 healthy controls. All recruited patients had a single-vessel critical coronary artery disease treated by primary angioplasty and by the placement of a drug-eluted stent; they also received the same drug therapy according to European Society of Cardiology (ESC) guidelines (aspirine, ticagrelor or prasugrel, beta-blocker, ACE Inhibitor and statin). In total, 30 patients were taught to meditate, 30 to appreciate music and 30 did not carry out any intervention and served as controls. Additionally, 30 healthy volunteers were also enrolled to rule out if the disease state could interfere with the relaxation effect, and 15 were trained to meditate and 15 were trained in music appreciation. Both meditation and music appreciation can induce the relaxation response (RR) in the same way [13]. Other details about the RR and the techniques that we used with the description of their pathophysiological mechanism is described in our previous works [11,12].

**Figure 1:**
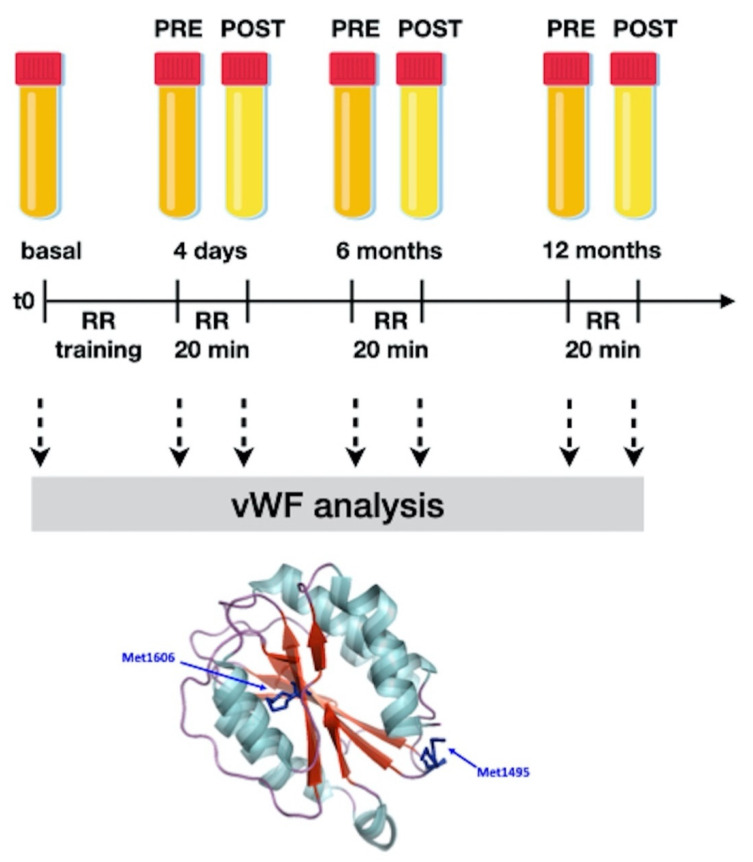
The study design—explanation is in the text. Relaxation response: RR. RR, 20 min: after 4 days of training, each subject relaxed through meditation or music appreciation for 20 min. A blood sample was taken immediately before (called PRE- in our work) and immediately after (POST- in our work) activity. The acute variation of the studied parameters can be attributed to the practice of relaxation according to the used methods as the precise timing of blood sampling (before and immediately after the end of the session) prevents any other influences. All groups were subjected to the same environmental conditions; the control patients were taken into our classroom for 20 min and were not subjected to any intervention. We simply asked them to relax and most of them sat down with their eyes closed. For more details, please see our previous works [11,12]. As a model, the crystallographic structure of the von Willebrand factor (vWF) A2 domain (PDB code 3GXB) is reported. The backbone is coloured according to secondary structure elements with α helices in light blue and β strands in red and loops in purple. The two representative methionine residues (i.e., Met1606 and Met1495) are shown in the stick representation. Images were generated with PyMOL vs. 1.3 (DeLano Scientific, San Diego, CA, USA).

After the initial four-day training, at 6 and 12 months of daily autonomous RR practice, a blood sample was taken immediately before and after the relaxation session (according to the scheme reported in Figure 1) to describe any acute variation of the markers that were object of this study (see Section 2.2).

**Figure 2:**
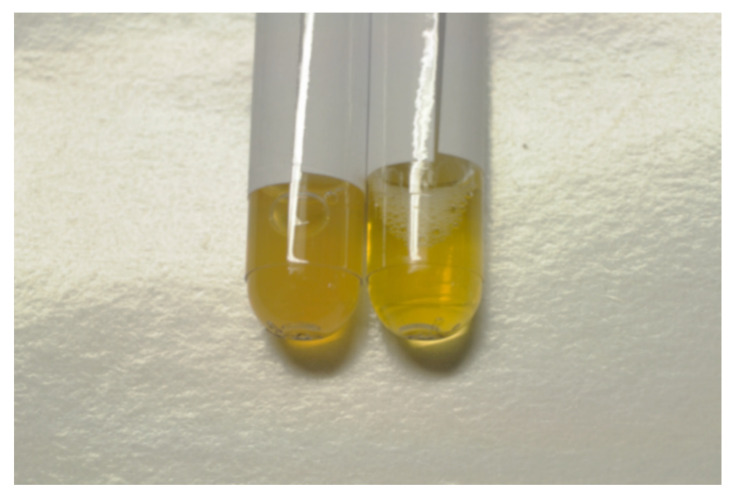
Variation of the physical characteristics of the plasma of the same patient after 20 min of meditation. On the left, the blood sample (after 4 min of centrifugation at 5000 rpm) before meditation is shown and is opalescent. On the right, the blood sample immediately after meditation is shown, which is clearer. The patient fasted for more than 5 h before meditating.

Clear variation of the physical characteristics of the serum samples (Figure 2) was observed.

As confirmed by prof. Benson’s researches [13,14] and our previous studies [11,12], the techniques employed in our work cause a similar relaxation effect without any significant difference. Therefore, all patients and healthy subjects treated with meditation and music were classified as, respectively, the “RELAXATION RESPONSE” group and the “RELAXATION RESPONSE HEALTHY CONTROLS” group. Finally, the patients that did not carry out any intervention constituted the “CONTROLS” group.

We emphasize that in our work we observed the RR using two conditioning techniques, meditation and music, which have to be considered in two ways to lead to the same relaxation effect [13,14]. Therefore, from a strictly methodological point of view we used a unique technique—precisely the RR—for which we needed to unite all the treated subjects in a single “intervention group”.

Indeed, all subjects enrolled in the study have continued the practice at home, twice a day, as they were taught. During the follow-up period, each subject reported having performed more than 80% of their meditation or music listening sessions. 

### 2.2. Determination of the Total Carbonyl Content

The carbonyl content of samples was measured using the Protein Carbonyl ELISA Kit (Abcam, Cambridge, UK), following the manufacturer’s instructions [15]. Briefly, carbonyls were quantified by reaction with dinitrophenylhydrazine (DNP), to form phenylhydrazone derivatives and were subsequent incubated with biotinylated anti-DNP antibody followed by horseradish peroxidase (HRP) conjugated secondary antibody. A calibration curve was obtained with BSA with a known content of carbonyl groups. The assay allowed us to obtain a reproducible sensitivity down to 10 pmol carbonyl/mg of protein, with an interassay variation of 10%. 

### 2.3. von Willebrand Factor Multimer Analysis

**Figure 3:**
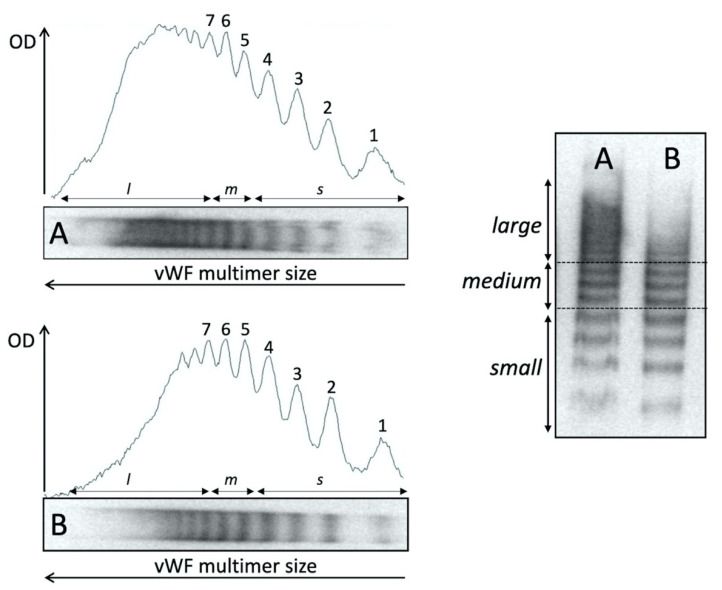
Densitometric analysis of vWF multimer pattern. Analysis of electrophoretic pattern was performed using ImageJ 1.52p software. Peaks 1 through 4 represent the small (s) multimers and peaks 5 and 6 represent the intermediate-medium (m) multimers. The large (l) multimers have peaks >7. OD, optical density. The dashed line indicates the limit of the tree different species of vWF multimers.

vWF multimers were analyzed by sodium dodecyl sulphate agarose gel electrophoresis combined with immunoblotting, chemiluminescent detection and densitometry analysis [16]. In detail, plasma samples were diluted in a loading buffer according to the amount of antigen and were subjected to nonreducing electrophoresis in the presence of an SDS denaturing agent in high gelling temperature agarose (1.6% Seakem HGT agarose; Lonza, Rockland ME, USA). Afterwards, the proteins were transferred by electroblotting to an immobilon polyvinylidene difluoride (PVDF) membrane (Millipore Corporation, Bedford, MA, USA) previously treated with methanol and distilled water. All incubation steps were performed in 5% low-fat milk. Visualization of vWF multimers was achieved by incubating the membrane with a polyclonal rabbit antihuman VWF antibody (Dako, Glostrup, Denmark), followed by a HRP-labelled goat antirabbit IgG (KPL, Gaithersburg, MA, USA). The immunodetection was performed using the Novex™ ECL chemiluminescent substrate reagent kit (Invitrogen, Carlsbad, CA, USA). vWF multimer gels were visually compared to a reference normal pooled plasma sample. Densitometric analysis was performed using ImageJ 1.52p software and the quantification of different vWF multimer sizes was carried out according to Studt JD et al. [17]. Briefly, one identified the typical triplet of vWF multimers from the end to the top of electrophoresis run, and they were classified into small (peak 1 to 4), medium (peaks 5 and 6), and large groups (LvWF, having a peak higher than 7). The percentages of the relative area values of each vWF multimer group were analyzed in comparison with the total integrated area of each lane, which was set as 100% and used for comparison between controls and RR samples (Figure 3).

### 2.4. Statistical Analysis

We assessed the basal and starting levels of vWF at every time point, which are expressed as percentages of the total median and interquartile range. The comparison between the pre–post intervention changes was performed using a Wilcoxon test calculating the percentage change of the markers during each follow-up. The comparison between groups was performed through the Mann–Whitney test. The distribution of the individual variables was assessed by the Shapiro–Wilk test. An initial comparison between groups was performed by means of Kruskal–Wallis test for independent samples or by Friedman test for paired data. Statistical significance was assumed if the null hypothesis could be rejected at *p* < 0.05. The statistical analysis was performed using software SPSS version 22.0 (Chicago, SPSS, Inc., Chicago, IL, USA). All conditions that could have affected the improvement of the serum molecules did not change and were comparable in the different groups at baseline, after 6 months and after 12 months (same physical rehabilitation and nutritional support, same therapy, same time of follow-up and same environmental conditions at the time of sampling). 

## 3. Results

### 3.1. Biochemical Markers

**Figure 4:**
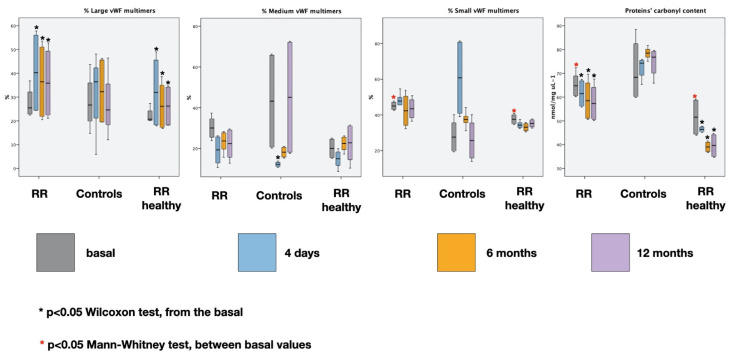
Basal and starting values of large, medium and small vWF percentages and total carbonyl contents at different time points. The median and interquartile range are also shown.

**Figure 5:**
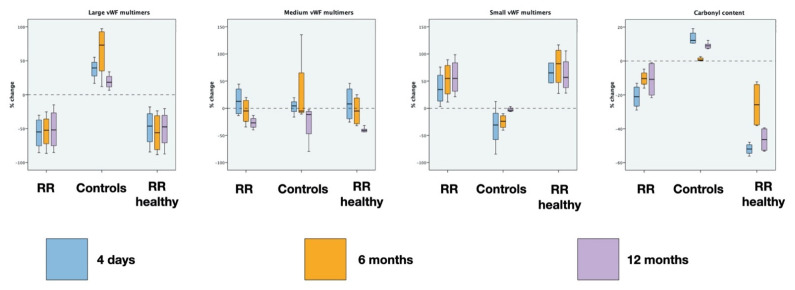
Markers’ percent change at different time points and median and interquartile range are shown. Explanation is in the text.

The results along with the statistical analysis of vWF multimers and total carbonyl contents are reported in Table 1, Table 2, Table 3 and Table 4 and in Figure 4 and Figure 5. 

As depicted in Figure 4, the starting values of LvWF multimers at every time point are increased from the basal (*p* < 0.05 Wilcoxon test) in both RR groups, while the starting total carbonyl content at every time point is decreased (*p* < 0.05 Wilcoxon test). The basal values (in grey) of small vWF multimers and carbonyl content are different between the RR group and RR healthy controls (*p* < 0.01 Mann–Whitney test—basal).

Figure 5 depicts the percentage variation of the markers during the 20 min sessions at every time point. The RR results in a significative decreasing of LvWF multimers (*p* < 0.001 Wilcoxon test at every time point), while in the controls they do not vary (*p* > 0.05 at every time point). On the contrary, small vWF multimers increase during RR at every time point (*p* < 0.01 Wilcoxon test at every time point) while no clear variations are appreciable in controls (*p* > 0.05 at every time point). No significative differences were detected for medium vWF multimers. 

The carbonyl content shows a significant decrease in case of RR and a significant increase in the controls (*p* < 0.01 Wilcoxon test at every time point).

It is possible to see that the behavior of the same markers is similar in individuals subjected to RR (*p* > 0.05 Mann–Whitney test at every time point, RELAXATION RESPONSE vs. RELAXATION RESPONSE HEALTHY CONTROLS).

### 3.2. Correlation between vWF Multimers and Total Carbonyl Content with Inflammatory Markers from Our Previous Studies

In our previous works we have shown how RR leads to a reduction in inflammation and body temperature together with an alkalinization of the pH [11,18]. Here, we consider IL6, TNF-alpha and TGF-beta as representative of the inflammatory molecules we tested in our previous studies in the same subjects due to their greater variation during the RR and their monotony trend. We pooled all the data before the RR and those collected immediately after to trace any correlation. Table 5 summarizes the medians of the above variables in our subjects [11,18].

A weak (nonlinear) correlation emerged (Table 6) between LvWF and small vWF multimers (SvWF), between medium (M vWF) and SvWF multimers and between LvWF multimers and pH, TNF-alpha and TGF-beta in both RR groups. In all the groups a weak correlation was present between TNF-alpha and total carbonyl content.

## 4. Discussion

Von Willebrand factor (vWF) is a multimeric plasma glycoprotein that plays a pivotal role in hemostasis and thrombosis, primarily by interacting with platelet adhesion receptors. vWF interacts with the platelet receptor GpIbα, initiating the transmembrane signaling events that ultimately results in the activation of the integrin αIIbβ3 activation and the platelet aggregation [19]. vWF is synthesized by vascular endothelial cells and megakaryocytes, and so-called ULvWF multimers are stored in endothelial Weibel–Palade bodies and platelet α-granules for later secretion [20,21]. Intermediate vWF multimers are circulating plasma species with molecular weights ranging from 3000 to 5000 kDa. These have low platelet binding affinity and seem to be FVIII carriers. After secretion, some ULvWF remains on the cell surface as very long strings that become decorated with platelets. Eventually, ULvWF multimers are converted into smaller, less thrombogenic fragments by the metalloprotease ADAMTS-13, which cleaves the Tyr1605-Met1606 bond in the central A2 domain of VWF [9,20]. Only vWF multimers with higher molecular weight shows a great propensity to interact with platelets, becoming prothrombotic and hemostatically active [21,22,23]. Thus, ULvWF are the only of clinical interest. Generally, intermediate vWF are not affect by proteolytic degradation and their distribution remain constant. Severe deficiency of plasma ADAMTS-13 activity (<5% of normal) is responsible for the persistence of hyperactive species of ULvWF on endothelial cells and in circulating blood [24]. Additionally, an imbalance of ADAMTS-13 activity and vWF plasma concentration are risk factors for the development of myocardial infarction, ischemic stroke, pre-eclampsia, malignant (or cerebral) malaria and antiphospholipid syndrome [25].

vWF plays important role not only in the coagulation equilibrium, but also in various nonhemostatic pathways such as apoptosis, angiogenesis, cell proliferation, and inflammation. During inflammation, vWF can participate in the recruitment of leukocyte regulating the exposition of P-selectin and may act as an important proinflammatory mediator of leukocyte extravasation [26]. Deficiency of vWF provokes impaired P-selectin surface expression and subsequent defects in leukocyte [27]. In addition, deficiency of ADAMTS-13 is associated with an increase in leukocyte rolling on unstimulated veins and, as a consequence, a major adhesion in inflamed vein [28]. Elevated vWF plasma levels have been described in diseases associated with systemic inflammation, such as chronic kidney disease [15], diabetes [29], and sepsis [30]. In these pathologic conditions, increased vWF plasma concentration independently correlates with increased risk of death. An important aspect of systemic inflammation is oxidative stress, caused by the oxidative burst in activated neutrophils, which subsequently release several reactive oxygen species (ROS) that include superoxide radical, hydrogen peroxide, and hypochlorous acid. In vitro evidence indicates that the oxidative stress generated during inflammation can abrogate ADAMTS-13 activity with the simultaneous ULvWF accumulation [31]. Since the interaction of vWF with GpIbα on platelets is predominantly governed by its multimeric size, this condition can lead to a prothrombotic state [30].

ROS oxidize cysteine and methionine residues one hundred times or more faster than the other amino acids, converting cysteine to cysteine sulfinic or sulfonic acids and methionine to methionine sulfoxide [28,29,30,31,32,33]. Interestingly, the A region of vWF contains 14 methionine residues, some of these are completely exposed (i.e., Met1495 in A2 domain), and other are buried in the hydrophobic area (i.e., Met1606 in A2 domain). Only methionine residues with the highest accessible surface, such as Met1495, are oxidized to a substantial extent under static conditions, in contrast only a conformational change imputable to shear stress can promote the oxidation of Met1606 [34]. It follows that A2 domain of vWF changes its conformation under shear stress, exposing the buried methionine and facilitating their oxidation. The crystallographic structure of the A2 VWF domain [35] shows that Met1606 is deeply buried, so the cleavage by ADAMTS13 and the possible oxidation can mediate only in the presence of chemical denaturants (i.e., urea and guanidine) and shear stress [34,36]. Notably, the oxidation of Met1606 only slightly increases the sidechain volume, while converts a hydrophobic amino acid into a polar and partially charged amino acid. The consequent drastic chemical perturbation introduced at position 1606 disrupts the continuous apolar surface, avoiding the interaction with ADAMTS13 [37].

We followed for one year some patients with ischemic heart disease and some healthy volunteers, after having taught them the relaxation response (RR) as part of a 4-day rational-emotional-education intervention and we documented a significant decrease in the perceived degree of stress combined with a reduction in different neuroendocrine-immune markers of inflammation and oxidative stress along with coronary endothelial function and flow improvements [11,12,38,39,40].

Based on the present results, analysis of vWF multimers and total plasmatic protein carbonyl content suggest that the RR may be accompanied by an antithrombotic state.

The protein carbonyl content is a stable biomarker of oxidative modification of proteins, that involved specifically amino acid sidechains (i.e., Pro, Arg, Lys, and Thr). The level of plasma protein carbonyls can positively correlate with ULvWF multimers expression and may be used as a surrogate marker of oxidative modification [29]. Although vWF is a minor component of plasma proteins; however, it is significantly sensitive to oxidative stress and undergoes oxidative modifications as in the case of much more abundant plasmatic proteins (i.e., albumin and fibrinogen). It is know that vascular inflammation and oxidative stress are linked with several chronic disorders, including hypertension, cardiovascular diseases, immunothrombosis, and aging [41]. For example, in the arterial wall, increases in inflammation and oxidative stress synergize to accelerate atheroma formation and increase risk of blood disease. RR therapy may prevent cardiovascular complications known to be induced by oxidative stress, in which vWF can be considered as a mediator of vascular inflammation. In this scenario, the prevention of vWF oxidation allows ADAMTS-13 activity to be maintained with a consequent reduction in UL-vWF secretion and impaired platelet aggregation and microvascular thrombosis. 

We have also shown a significant reduction in body temperature during RR (about 0.2–0.5 centigrade degrees) [11], a variation in pH (alkalinization) and a reduction in the electrical conductivity of the subjects’ serum [18].

This could be very important because conformational transition of vWF is also modulated by its thermodynamic state, that, in turn, is determined by physical (e.g., vessel geometry), physico-chemical (e.g., pH) and molecular-biological (e.g., ions and binding molecules) factors [42]. vWF globule-stretched transition varies with pH, calcium and other ions, temperature, binding of albumin, etc., resulting in a dynamic state-diagram of vWF [42]. vWF keeps attached in a globular conformation on endothelial cell surface upon acidic conditions but gets elongated and released upon realkalinization [42]. The mild correlation between L vWF multimers, pH and inflammatory markers seems to be in line with this.

A certain physical state is associated with a certain physiological function. At high extension and force, for instance, VWF is rendered adhesive, while the opposite (small extension and force) creates a rather inactive state. 

## 5. A Possible Underlying Mechanism According to a Biophysical Perspective: A Molecule Conformational Change and Plasma Coherent States

Biological systems are complex systems operating at a multitude of interdependent levels, from molecules to macromolecules and cells, from cell aggregates to macro-organisms and ecosystems [38], with an intricate net of feedback flows of electrochemicals mediators. The unitary functional activity of the system is ensured by dynamical laws of coherence generating long-range correlations of the system components. 

Biomolecules and the water molecules of their embedding bath are physically characterized by their molecular electric dipoles. Randomly oriented dipoles have spherical symmetry, which may be broken by the polarizing action of an electric field due to ions, molecular aggregates or some external agents. The electrical polarization *P* will then characterize the state of the oscillating dipoles, pointing now in the average along the direction of the applied electric field. *Polarization waves* thus span the system (*collective modes*). They are not destructively interfering among themselves and therefore are called *coherent waves* [42,43,44]. It is their *coherence* that allows the possibility of the nonzero polarization density *P*(*x*,*t*). In the noncoherent case, the polarization is zero. *P*(*x*,*t*) provides a measure of the coherence and is named *order parameter* since it describes the nonrandomly oriented (ordered) oscillating dipoles. 

Of course, the system state with *P*(*x*,*t*) ≠ 0 has *macroscopic* properties different from the state with *P*(*x*,*t*) = 0. Coherent waves among *microscopic* components thus determine the *macroscopic* properties of the system.

Any change in the value of *P*(*x*,*t*) describes a *phase transition*—namely, the *rearrangement* in the coherent net of long-range correlations, and thus a *different dynamical regime* of the oscillating dipoles. Phase transitions are typically accompanied by *critical* processes—namely, discontinuities in relevant parameters and/or conformational changes in the system components. 

The many-body model predicts [38,43] that RR practice, which appears to promote healthy metabolic and organizational biological activity [13], is favorable to the dynamical formation of coherent long-range correlations among electric dipoles of macromolecules and plasma (water) molecules. Perturbations of the coherent dipole ordering may instead negatively affect healthy biochemical activity, leading to, e.g., oxidative stress or else functional unbalances [44], body temperature reduction during RR [11], variations in pH and electric conductivity [18] and increased transparency of the plasma. 

The vWF molecular processes offer a further example consistent with such a model. 

From the results of previous sections, we see that the possibility of vWF being involved in the biochemical activity depends on conformational changes induced by friction shear stress of its chemical domains due to surrounding molecular flow. The resulting changes of vWF polar properties produce a chain of consequent steps, perturb its interaction with metalloprotease ADAMTS-13 [37], which then can cleave the Tyr1605-Met1606 bond in the A2 domain [9,24]. This favors the exposition of Met1606 to oxidation, and thus a hydrophobic amino acid is changed into a polar and partially charged one. 

We thus realize the possible explanatory link between the higher functional levels activated in the RR practice and the observed decrease in large vWF multimers. The mentioned vWF conformational changes, with the resulting polar changes from the one side, and the coherent dipole long-range correlations in the plasmatic environment from the other side, enter into a circular, nonlinear chain of reciprocal interactions with possible self-regulatory back-reaction effects. 

Moreover, they are nonlinearly dependent on several physical factors (e.g., temperature) and chemical factors (e.g., the density of ULvWF in the plasma), which determine the dynamical criticality mentioned above. 

A further remark is on vWF and blood flow vorticity. Once originally triggered by the heart contraction and torsion motion, the persistence of the vorticity of the blood flow propagation depends on the contribution to its viscosity by its molecular components [44], such as, e.g., aggregates of platelets. We then realize the relevance of vWF multimers due to their role in promoting the aggregation of platelets. The vWF contributes to the control of coagulation processes and thus the critical balancing of laminar and vorticose blood flow.

In conclusion, the psychic activity appears to be linked to molecular processes by controlling the density of biochemical agents in the plasma and by triggering molecular rearrangements (such as the fragmentation of ULvWF).

## 6. Study Limitations

The basal values of the markers over time may be influenced by other factors (other than RR) such as alimentation, physical activity, smoking, different levels of stress between people, etc.

RR is a pleiotropic response that can have differences between people and that can “succeed” to different extents. In any case, the precise study design contains this bias as each subject is compared with himself and the trend of the variables was confirmed three times.

Regarding the correlation between variables, however “chemically” logical, they are questionable at the “biophysical” level. Each studied molecule oscillates and changes its spatial conformation, place and quantity continuously. We have arbitrarily observed a single “frame” of this dynamic “film” three times, in a “single place” (the site of the blood sampling) in the same way. However, the “frame” is not the “movie”. The variables object of this research (and not only) may be linked over time (in physics the term “*coherent*” is used) even if not punctually or instantly “statistically correlated”.

The analysis and results presented above confirm that molecular components of biological systems play of course a crucial role as chemical agents in complex chemical processes and pathways and, at same time, they play an equally crucial role of a physical nature, as “nodes” in a pervading net of *coherent correlations* describing *collective modes*, which characterize the mesoscopic and macroscopic functional activity of the system. These two roles, the chemical one and the physical one, played at once by the same microscopic agents, differ in the range of action of their respective couplings. One of them is a *short-range* coupling typical of chemical interactions, localized over limited molecular regions. The other one is a *long-range* correlation spanning over domains of size much wider than the single component dimensions. This last one is of a dynamical resonant nature (*in phase* coupling) and produces regulatory actions on the chemical activity in highly nonlinear forward–backward processes minimizing free energy and thus ensuring the system functional activity against (unavoidable) microscopic fluctuations in the chemical activity. Of course, when focusing on the molecular chemical role, the collective modes remain unobservable; they disappear when the system is split into its chemical components. This is similar to what happens in condensed matter physics where, the system properties, e.g., the crystal properties, cannot be seen when studying the individual component atoms. Moreover, in physics as well as in biology, functional properties develop in time in a complex way and one cannot pretend to reconstruct “the movie” from the observation of isolated frames or short sequencies. Unfortunately, these limitations also apply to our stud y. Further analysis is thus necessary and is part of our future plans.

## Figures and Tables

**Table 1:** Large vWF percentage, medians and interquartile range; basal values (grey); after 4 days of RR training (blue), after 6 (orange) and 12 (violet) months of RR practice. PRE means before and POST means after the RR practice session. In the row under each group the % change observed within each follow-up session is shown.

Groups	Large vWF Basal	Large vWF 4D PRE	Large vWF 4D POST	Large vWF 6M PRE	Large vWF 6M POST	Large vWF 12M PRE	Large vWF 12M POST
Relaxation Response	24.95 (22.4–27.5)	40.24 (24–56.1)	12.6 (8.2–17)	36.42 (21.8–51)	15.25 (6.9–23.6)	35.85 (22–49.3)	14.85 (7.3–22.4)
% change		−54.85 (−70.38–−40.83)		−52.37 (−65.31–−41.18)		−51.9 (−70.19–−32.7)	
Controls	25.17 (22.55–29.8)	36.4 (28.78–39)	48 (44.08–51)	32.25 (19.5–45)	40 (34–40.4)	24.6 (21.4–30)	31.65 (26.2–39)
% change		39.38 (32.95–44.06)		73.09 (46.51–90.43)		18.27 (14–23.46)	
Relaxation Response Healthy Controls	20.7 (20.3–21.1)	31.95 (18–45.6)	11.05 (7.1–15)	26.11 (17–35.02)	8.6 (4.1–13.1)	26.2 (18–34.2)	10.3 (4.3–16.3)
% change		−46.23 (−61.93–−33.03)		−56.06 (−77.45–−34.68)		−47.43 (−62.68–−35.44)	

**Table 2:** Medium vWF Percentage, medians and interquartile range; basal values (grey); after 4 days of RR training (blue), after 6 (orange) and 12 (violet) months of RR practice. PRE means before and POST means (after) the RR practice session. In the row under each group the % change observed within each follow-up session is shown.

Groups	Medium vWF Basal	Medium vWF 4D PRE	Medium vWF 4D POST	Medium vWF 6M PRE	Medium vWF 6M POST	Medium vWF 12M_PRE	Medium vWF 12M POST
Relaxation Response	30.05 (25.5–34.6)	19.3 (12.9–25.7)	23.2 (11.6–34.8)	23.7 (19.7–27.7)	23.3 (14.9–31.7)	22.4 (15.7–29.1)	16.9 (10.2–23.6)
% change		12.67 (−10.08–35.41)		−4.96 (−24.37–14.44)		−26.97 (−35.03–−18.9)	
Controls	43.25 (31.9–54.3)	13.6 (12.7–13.9)	14.5 (13.2–14.8)	26.7 (19.4–32.8)	31.1 (26.8–35.4)	31.4 (28.1–41.6)	23.05 (15.4–30.5)
% change		4.32 (−0.82–8.12)		−5.18 (−6.58–29.97)		−8.58 (−30.62–−2.87)	
Relaxation Response Healthy Controls	20.05 (15.5–24.6)	15 (11.7–18.3)	17.1 (9.4–24.8)	22.5 (19.4–25.6)	22.1 (13.8–30.4)	22.8 (14.5–31.1)	13.65 (8.2–19.1)
% change		7.93 (−19.66–35.52)		−5.06 (−28.87–18.75)		−41.02 (−43.45–−38.59)	

**Table 3:** Small vWF percentage, medians and interquartile range; basal values (grey); after 4 days of RR training (blue), after 6 (orange) and 12 (violet) months of RR practice. PRE means before and POST means after the RR practice session. In the row under each group the % change observed within each follow-up session is shown.

Groups	Small vWF BASAL	Small vWF 4D PRE	Small vWF 4D POST	Small vWF 6M PRE	Small vWF 6M POST	Small vWF 12M_PRE	Small vWF 12M POST
Relaxation Response	45 (43–47)	47.75 (45.6–49.9)	64.25 (48.3–80.2)	42.3 (34.1–50.5)	61.45 (44.7–78.2)	43.45 (38.4–48.5)	65.15 (54–76.3)
% change		34.54 (18.21–53.38)		54.91 (33.99–73.58)		55.02 (36.34–76.2)	
Controls	27.55 (23.53–31.58)	49.7 (47.48–57.48)	34.4 (28.9–37.3)	43.5 (38.4–47.7)	30.25 (28.5–35.58)	39.75 (30.58–44)	42.9 (41.5–44)
% change		−30.78 (−44.22–−19.96)		−24.03 (−32.75–−15.89)		−3.5 (−4.89–−1.07)	
Relaxation Response Healthy Controls	37.5 (35–40)	34.25 (32.9–35.6)	56.8 (48.3–65.3)	33.1 (31.1–35.1)	56.05 (44.7–67.4)	35.25 (33.2–37.3)	56.15 (44–68.3)
% change		65.12 (46.81–83.43)		82.04 (57.35–101.72)		56.84 (42.96–75.72)	

**Table 4:** vWF carbonyl content (median and interquartile range). Values after 4 days of RR training (blue), after 6 (orange) and 12 (violet) months of RR practice. PRE means before and POST means after the RR practice session. In the lower rows of the table the % change observed within each follow-up session is shown.

Groups	Carbonyl Basal	Carbonyl PRE	Carbonyl POST	Carbonyl PRE6	Carbonyl POST6	Carbonyl PRE12	Carbonyl POST12
Relaxation Response	64.72 (60.56–68.88)	61.44 (56.08–66.8)	48.8 (41.1–56.48)	58.48 (51–65.92)	52.16 (47.4–56.88)	57.3 (50.5–64.08)	51.72 (40.31–63.12)
Controls	68.28 (60.15–79.42)	74.24 (71.1–75.44)	83.36 (81.1–84.26)	78.48 (77.62–79.28)	78.56 (77.86–79.86)	76.76 (72.2–79.2)	83.64 (77.82–87.04)
Relaxation Response Healthy Controls	51.55 (44.33–58.77)	46.49 (46.32–46.66)	22.34 (21.24–23.43)	39.03 (37.02–41.03)	29.19 (23.05–35.32)	39.63 (35.07–44.19)	20.96 (20.89–21.03)
	% change			% change		% change	
Relaxation response	−21.06 (−26.68–−15.45)			−10.38 (−13.71–−7.05)		−10.85 (−20.21–−1.5)	
Controls	12.17 (10.5–15.15)			0.53 (0.1–1.36)		8.93 (7.77–9.9)	
Relaxation response healthy controls	−51.95 (−54.48–−49.42)			−25.83 (−37.74–−13.92)		−46.38 (−52.73–−40.03)	

**Table 5:** Body temperature, pH, IL-6, TNF-alpha, and TGF beta values of each group are shown. For all the methodological issues and more detail please see our previous studies [11,18].

Groups	Body Temp.		pH		IL-6 ng/L		TNF-alpha pg/mL		TGF-beta μg/L	
	PRE	POST	PRE	POST	PRE	POST	PRE	POST	PRE	POST
RELAXATION RESPONSE	36.6 (36.1–36.9)	36.3 (35.9–36.5)	7.0 (6.7–7.3)	7.4 (7.2–7.7)	3.7 (2.9–4.3)	2.6 (<2–3.1)	4.5 (2.8–5.4)	2.9 (<2–3.6)	37.9 (35.9–43.8)	31 (26.4–38.2)
CONTROLS	36.5 (36.1–36.7)	36.7 (36.3–36.9)	7.4 (7.2–7.6)	6.9 (6.7–7.3)	3.6 (2.8–4.0)	4.1 (2.6–4.3)	4.3 (2.6–5.6)	5.9 (2.8–6.1)	36.3 (35.4–38.7)	42.7 (39.5–43.9)
RELAXATION RESPONSE HEALTHY CONTROLS	36.4 (35.9–36.7)	36.2 (35.7–36.4)	7.3 (7.0–7.4)	7.5 (7.3–7.5)	2.8 (<2–3.2)	<2 (<2–2.4)	2.4 (<2–3.5)	2.0 (<2–2.6)	32.5 (26.5–39.8)	28.5 (21.7–36.7)

**Table 6:** Correlation matrix: Spearman coefficient (bold font represents values with *p* < 0.05).

*RELAXATION RESPONSE*									
	***L vWF***	***M vWF***	***S vWF***	***Carbonyl***	***Body Temp***	***pH***	***IL6***	***TNFalpha***	***TGFbeta***
**L vWF**	1								
**M vWF**	0.4488212	1							
**S vWF**	**−0.8031173**	**−0.7695195**	1						
**Carbonyl**	0.54983667	−0.0934422	−0.2273933	1					
**Body Temp**	0.4951998	−0.1058215	−0.1972022	0.13290312	1				
**pH**	**−0.7568174**	0.1611795	−0.0724475	0.02068901	−0.7098822	1			
**IL6**	0.1643193	−0.0530246	−0.0165857	0.22071129	0.35427728	−0.0521386	1		
**TNFalpha**	**0.6553398**	0.24696555	−0.1065792	**0.73033795**	−0.6644612	0.45628284	−0.1281666	1	
**TGFbeta**	**−0.7298136**	−0.2302344	0.28479838	−0.1833968	0.08288911	0.21092171	0.74492299	−0.1522784	1
*CONTROLS*									
	***L vWF***	***M vWF***	***S vWF***	***Carbonyl***	***Body Temp***	***pH***	***IL6***	***TNFalpha***	***TGFbeta***
**L vWF**	1								
**M vWF**	0.24257875	1							
**S vWF**	−0.5665193	−0.1410782	1						
**Carbonyl**	−0.5280698	−0.1795384	0.08943854	1					
**Body Temp**	0.59709917	0.0075333	−0.1325308	−0.4643379	1				
**pH**	0.01243143	0.23315709	0.25956481	−0.2490491	0.120824	1			
**IL6**	−0.0134115	0.21426733	0.06986446	0.06005788	−0.0399083	−0.5057946	1		
**TNFalpha**	0.13471092	−0.3923573	−0.1826808	**0.7773945**	0.10633633	−0.3915101	0.14474083	1	
**TGFbeta**	0.24206284	0.09167871	−0.1731709	−0.355089	−0.1506871	−0.01284	−0.0760909	0.52394815	1
*RELAXATION RESPONSE HEALTHY CONTROLS*									
	***L vWF***	***M vWF***	***S vWF***	***Carbonyl***	***Body Temp***	***pH***	***IL6***	***TNFalpha***	***TGFbeta***
**L vWF**	1								
**M vWF**	0.735540032	1							
**S vWF**	**−0.7075044**	**−0.7317979**	1						
**Carbonyl**	−0.2572203	−0.1531874	0.5644646	1					
**Body Temp**	0.15639389	−0.0084742	−0.2854408	−0.0220424	1				
**pH**	**−0.70486633**	−0.2336897	−0.0158521	−0.4094433	0.35116455	1			
**IL6**	−0.1155365	0.0595067	0.04987164	0.16705885	0.07275114	−0.024814	1		
**TNFalpha**	**0.6551395**	−0.3787234	0.31082905	**0.75718815**	0.21356039	0.55018354	−0.0013061	1	
**TGFbeta**	**−0.6993715**	−0.2250648	0.61388811	0.2342013	0.00280797	0.19113802	0.36220604	−0.6143749	1

## Data Availability

All data are contained within the article.
